# Variability of the key features and revision of a group of closely related species of grassflies (Diptera, Chloropidae, *Meromyza*)

**DOI:** 10.3897/zookeys.942.49644

**Published:** 2020-06-18

**Authors:** Andrej F. Safonkin, Aleksandra A. Yatsuk, Tatiana A. Triseleva

**Affiliations:** 1 Severtsov Institute Ecology and Evolution RAS, Leninsky av., 33, Moscow, 119071, Russia Severtsov Institute Ecology and Evolution RAS Moscow Russia

**Keywords:** CO1 mtDNA, meigeni group, morphological features, postgonite, variegata group

## Abstract

The following external morphological features of members of the genus *Meromyza* Mg. have been analyzed: the ratio of the height of frontal triangle to the length of the head; the presence of black setae on the lower surface of genae; the degree of manifestation of ocellus spot; the ratio of the length of mesonotum to scutellum; the length and color of the mid strip of the mesonotum and its degree of manifestation on the scutellum; and the thickness of hind femurs. Additionally, the size and shape of anterior and posterior processes of the postgonite, and the type of attachment of the posterior process have been investigated. The authors have determined the range of variability of key features applicable to the reliable identification of closely related species of grass flies in the “meigeni” species group of the genus *Meromyza*, as well as the usefulness of CO1 mtDNA sequences for this purpose. The authors propose to re-instate the name *M. laeta* Meigen, 1838 (instead of using the name *M. meigeni* Nartshuk, 2006), to include seven species into the cluster “meigeni”, and to substitute the name of the cluster “meigeni” with the name “variegata”.

## Introduction

The high variability of key external morphological features of *Meromyza* flies (Diptera, Chloropidae, *Meromyza* Meigen, 1830) has been noted by many authors (Nartshuk and Fedoseeva 2010). High variability in the color of palpi and mid stripe of the mesonotum, and shape of ocellus and occipital spots, etc. make it difficult to precisely identify specimens. Before features of the genital apparatus became key to the identification of *Meromyza* flies, only six species of this genus had been described, and species synonymy was highly disputed (Fedoseeva 1960). To date, *Meromyza* includes more than 90 species, and the identification key is based on a combination of external morphological features, and specific features of male genital apparatus (Nartshuk and Fedoseeva 2010). Recently, it was shown that the precision of species identification can be verified using molecular-genetic analysis of CO1 mtDNA gene (Safonkin et al. 2016). The results of genetic analysis combined with the shape of the anterior process of the postgonite made it possible to divide the genus into eight clusters, which were named after the species closest to the hypothetical ancestor (Safonkin et al. 2016). The flies of the “meigeni” cluster are populous throughout Europe. Excessive variability of external features in females of the “meigeni” cluster makes species identification based on female features very difficult. Despite the similarity of male anterior and posterior processes of the postgonite, the degree of their variability and its usefulness for species identification has not been previously studied. Upon our findings, the larvae of *Meromyza variegata* Meigen, 1830 from this cluster damage oat shoots, which necessitates the correct identification of adults.

The aim of this study was to determine the variability of key features of “meigeni” cluster flies, to select features unique for valid identification of the species, and to re-examine the naming of this cluster.

## Material and methods

Collection sites of the material. *Meromyza* species were acquired from the collection of the Severtsov Institute of Ecology and Evolution (Moscow, Russia), and comprised material collected in different years in the Moscow, Tver and Tula regions of the Russian Federation, as well as the Brest region of the Republic of Belarus. In 2018, we also collected flies from the Czech Republic in the Pilsen Region (June 20, 49°75'82"N, 13°15'61"E), Jihlava (June 23, 49°39'66"N, 15°59'96"E), Brno (June 24, 49°23'01"N, 16°53'33"E) and Olomouc (June 25, 49°63'40"N, 17°34'35"E). We used *M. bohemica* Fed. as a model for the analysis of population variability of morphological features in the “meigeni”cluster.

External key features. Based on our own and published data, we analyzed face profile, the shape of the 3rd antennal segment, the length of the head, the color of arista and palpi, the height and width of the frontal triangle and their ratio, the ratio of the length of the head to the height of frontal triangle, the ratio of the genae height to the height of the 3rd antennal segment, the wrinkledness of the apex of frontal triangle, the shape and size of the ocellus spot, the presence of black setae on the lower surface of genae, the parafacial angle; the pattern of the occiput; the length of mesonotum and scutellum and their ratio, the length of the mid stripe of mesonotum, the color and length of mesonotal stripes, the presence of a stripe on scutellum, the color of the abdomen, the thickness of hind femurs, and body length.

Postgonites. The shape of the postgonite was studied using images acquired with a CamScan MV 2300 scanning electron microscope (Czech Republic). Measurements of the lateral surface of the postgonites for 80 *Meromyza* specimens were carried out using images acquired by a Keyence VHV-1000 light microscope (Japan), with an integrated data analysis program and with standard settings used throughout the study. We investigated a shape and an area of the anterior process of the postgonite, and the type of attachment of the posterior process. Measurements were recorded in micrometers. To prepare the samples, we extracted the postgonites from the abdomen of flies and affixed them onto paper.

We performed molecular-genetic analysis based on the nucleotide sequences of CO1 mtDNA locus previously obtained and deposited by us in GenBank (Safonkin et al. 2016). We deposited new nucleotide sequences of CO1 mtDNA from *Meromyza bohemica* Fedoseeva, 1962, *M. femorata* Macquart, 1835 and *M. rufa* Fedoseeva, 1962 in GenBank with accession numbers MN 037808–MN 037814. The construction of the phylogenetic tree with new nucleotide sequences and analysis of phylogenetic relations were performed using the MEGA5 (Tamura et al. 2011) software package. Statistical data analysis was performed using Statistica 10.

## Results

Species differences of the cluster “meigeni”. Based on the analyzed features (see methodology), most features either do not differ, or their dimensional boundaries overlap. We determined that the following features are the most applicable for species identification (Table 1): 1) The head length and the height of the frontal triangle are maximal in *M. femorata* and minimal in *Meromyza mosquensis* Fedoseeva, 1960; 2) Black setae on the lower surface of genae are found in *M. bohemica* and *Meromyza elbergi* Fedoseeva, 1979, and sometimes in *M. femorata* and *M. variegata*; 3) A dark occipital spot is observed in *M. mosquensis* and *M. elbergi*, and there are light lateral occipital stripes in some species (only *M. bohemica* has brown strips); 4) The length of the mesonotum and scutellum and their ratio are maximal in *M. meigeni* Nartshuk, 2006, and minimal in *M. femorata*; 5) The stripe of the mesonotum reaches the scutellum in *M. meigeni* and passes through the scutellum in *M. mosquensis* and *M. elbergi*; 6) The darkened part of the mesonotal mid stripe varies slightly in all species and differs significantly in color (*M. femorata* and *M. rufa* have lighter stripes); 7) *M. mosquensis* and *M. elbergi*, and occasionally *M. femorata*, have a marked stripe on the mesonotum; 8) The thickness of hind femurs significantly varies among species.

**Table 1.** Characteristics of key features of the species in the proposed “variegata” cluster of grassflies *Meromyza* according to present and literature data; *N* - number of specimens measured, * only literature data.

Characteristics	*M. bohemica N* = 9	M. elbergi*	*M. femorata N* = 11	*M. laeta* (*M. meigeni*) *N* = 18	*M. mosquensis N* = 13	*M. rufa N* = 3	*M. variegata N* = 11
Length of the head, mm	0.587±0.019	–	0.607±0.017	0.487±0.016	0.467±0.010	0.524±0.004	0.570±0.023
Height of frontal triangle, mm	0.376±0.013	–	0.419±0.012	0.329±0.112	0.313±0.009	0.320±0.008	0.380±0.024
Ratio of the genae height to the height of the 3rd antennal segment	0.83±0.02	1.5	1.03±0.05	0.77±0.06	0.75±0.04	0.64±0.03	0.74±0.04
Setae and the bristles on the lower surface of genae	light with some black setae	many black setae	black or white	light	light	light	light sometimes black
Color of the palpi	light	black in distal part	black in distal part	black	light	light	light
Occiput pattern (spot/lateral strips)	no/brown	brown/ brown, sometimes the occeput is dark	no/no	not intensive/ not bright	dark /dark, sometimes the occeput is dark	no or not intensive/ yes	not intensive/ not bright
Length of mesonotum, mm	0.916±0.018	–	0.947±0.037	0.769±0.017	0.762±0.020	0.742±0.029	0.898±0.039
Length of the scutellum, mm	0.305±0.008	–	0.323±0.012	0.218±0.006	0.225±0.007	0.222±0.012	0.288±0.008
Ratio of mesonotom to scutellum	3.01±0.06	–	2.93±0.06	3.54±0.11	3.39±0.06	3.37±0.32	3.11±0.07
Stripe of the mesonotum passes through the scutellum	no	yes, broad	no	sometimes	yes, broad	no	no
Proportion of colored part of the mid stripe of the mesonotum (%)	72.9±2.1	–	73.3±2.5	69.4±1.3	100	72.1±2.7	73.1±2.3
Color of the mid and lateral stripes	brown, light brown, black outer margins of lateral stripes	black, sometimes brown	reddish, rich red, yellow, sometimes brown	brown dominates over black, lateral strips are often black, sometimes all stripes are brown or yellow	brown to black	rust-colored, brown, yellow-brown, black outer margins of lateral strips	brown, sometimes black, lateral strips are darker with black margins
Ratio of hind femurs to hind tibia	3.27±0.11	almost three times over	4.13±0.23	3.19±0.26	3.03±0.12	3.83±0.20	3.80±0.15
Length of the body, mm	3–3,5	3.5–4.5	4.5–5	3–3.5	3–3.5	3.5–4	4–5

Males differ in the structure and size of the postgonites. The difference in the area of the anterior process of the postgonite is statistically significant in most species (Table 2). The species also differ in shape of the anterior and posterior processes of the postgonite, and by the type of attachment of the posterior process to the anterior one, and by the line of attachment of the posterior process relative to the line of attachment of the anterior process of the postgonite to the hypandria.

**Table 2.** Characteristics of the postgonite of the proposed “variegata” species of grassflies *Meromyza*.

Characteristics	*M. bohemica*	*M. femorata*	*M. laeta (M. meigeni)*	*M. mosquensis*	*M. rufa*	*M. variegata*
Attachment of posterior process of the postgonite to the anterior one	laterally	posterior	posterior	posterior	posterior	posterior
Line of attachment of posterior process of the postgonite to the anterior one	above	down	almost down	above	above	almost down
Shape of posterior process of the postgonite	acuminate, slightly curved forward	acuminate, slightly curved forward	acuminate, curved forward	not acuminate, curved forward	round-ended, getting broader downward	acuminate, curved forward
Tip of the anterior process of the postgonite	sharply stubbed, acuminate	acuminate, Stubbed, transverse folds	oval	slightly stubbed, oval	obtusely stubbed	diagonally stubbed
Area of anterior process of the postgonite, µm² (n specimens)	4365.0±139.4(9)	7228.7± 93.1 (23)	4512.6±91.6 (4)	5507.9± 87.0 (22)	3053.8±296.3 (3)	9010.2±134.3 (20)

The population variability of key features was analyzed in *M. bohemica* Fed. as the model species. The first feature was the black setae on the lower surface of genae; a 10% and 15% variability in number of individuals with more than five setae was observed in the same population, and among studied populations, respectively.

The second feature was the length of the mid stripe of the mesonotum. In most adult flies, the length of the stripe was ¾ the length of the mesonotum. The largest proportion of males and females with extreme length ratios (2/3 and 1) were found in the eastern population.

**Table 3.** The number of setae and the proportion of specimens (%) with a large number of setae (more than five) on the lower surface of the genae in males and females in four populations of *M. bohemica*: number of specimens (*N*), number of setae <5 or >5 (fewer or more than five).

Collection sites, percent of flies with more than 5 setae (%>5)	Side of the genae	Males				Females				Population Average %
		*N*	<5	*N*	>5	*N*	<5	*N*	>5	>5
Plzen	right	70	2.2±0.2	24	6.4±0.4	42	1.7±0.2	23	6.0±0.4	
	left		2.1±0.2		6.6±0.5		2.2±0.3		6.3±0.4	
% >5					25.5				35.4	30.5
Jihlava	right	38	2.1±0.2	38	6.1±0.6	40	2.3±0.2	10	6.7±0.4	
	left		2.0±0.2		6.2±0.6		2.4±0.3		5.9±0.6	
% >5					19.1		20.0			19.6
Brno	right	91	2.0±0.2	20	5.5±0.4	67	1.9±0.2	11	5.5±0.3	
	left		2.0±0.1		5.4±0.4		2.1±0.2		5.7±0.4	
% >5					18.0				14.1	16.1
Olomouc	right	30	2.1±0.3	11	5.5±0.4	21	1.6±0.2	10	5.6±0.4	
	left		1.8±0.3		5.5±0.4		1.5±0.3		5.8±0.3	
% >5					26.8				32.3	29.5
Average	right	229	2.1±0.1	64	5.9±0.2	170	1.9±0.1	54	6.0±0.2	
	left		2.0±0.1		6.0±0.3		2.1±0.1		6.0±0.2	
% >5					21.8				24.1	23.9

The third feature was the color of the mid stripe of the mesonotum. Red is the most common color, with greater number of specimens with dark red or brown stripes found in the central and western populations.

**Figure 1.**
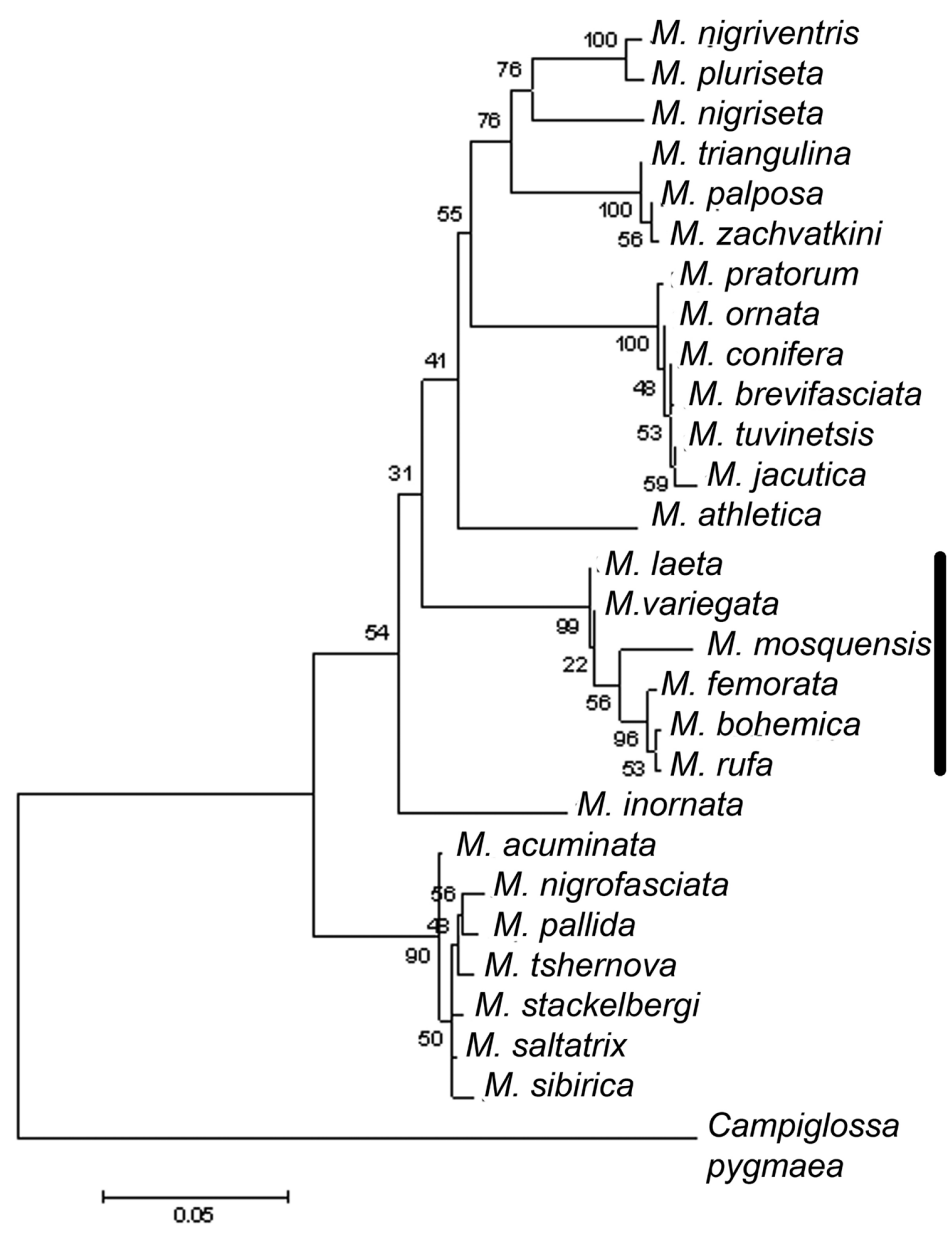
Maximum-likelihood phylogenetic tree showing relationships between the species of the “variegata” cluster and other species of *Meromyza* flies, based on CO1 mtDNA locus. The bootstrap values are given at the nodes. Vertical line – cluster “variegata”. *M. laeta* Fedoseeva, 1960 = *M. meigeni* sensu Nartshuk, 2006. The scale bar shows the genetic distances between the haplotypes. The outgroup was *Campiglossa pygmaea* Novak, 1974 (Diptera, Tephritidae) (GenBank: HM062547.1).

The phylogenetic tree based on the CO1 mtDNA of previously obtained nucleotide sequences (Safonkin et al. 2016) and new ones from *M. bohemica*, *M. femorata* and *M. rufa* puts the sequences from these species into the cluster composed of *M. meigeni*, *M. mosquensis*, *M. variegata*; *M. meigeni* and *M. variegata* are the most close to a hypothetical haplotype of the cluster, also we can easily see that other species of this cluster divide from *M. variegata* (Fig. 1).

## Discussion

The first attempt to divide 28 new species of *Meromyza* into four distinct groups based on the male’s postgonite morphology was made by Hubicka (1970). Eight species, *M. rufa*, *M. bohemica*, *M. lolli* Hubicka 1967, *M. eduardi* Hubicka, 1966, *M. femorata*, *M. laeta* Meigen, 1838, *M. variegata*, and *M. stackelbergi* Fedoseeva, 1967, were placed by this author into a separate group (“variegata”). Our comparative analysis of key features described by Hubicka (1970) for species of this group revealed that postgonite morphology of *M. eduardi* and *M. stackelbergi* differed sharply from the other six species in this group which cannot be reliably distinguished by external morphological features. Three of the species from the “variegata” group, *M. bohemica*, *M. lolli*, *M. femorata*, had black setae on the lower surface of genae, but three others, *M. laeta*, *M. mosquensis*, *M. rufa* and *M. variegata*, did not have this feature. Based on the analysis of population variability of *M. bohemica*, we concluded that this feature is not unique. Also, our comparative analysis of population variability in occipital stripes and the color range of the stripes of the mesonotum in *M. bohemica* and *M. lolli*, considered by Hubicka (1970) as key features of species in the “variegata” group, showed that these morphological features are also non-unique. This fact is confirmed by the most complete key feature tables (Fedoseeva 2003, Nartshuk and Fedoseeva 2010), in which *M. lolli* is absent. In our opinion, five species from the “variegata” group described by Hubicka (1970) can be included in a separate cluster, previously designated by us as “meigeni” (Safonkin et al. 2016). The difficulty of identification of species of the considered group is confirmed in the case of *M. lidiae* Nartshuk, 1992. This species, according to Nartshuk (1992), is close to *M. laeta*. However, Nartshuk and Fedoseeva (2011) pointed out that *M. lidiae* is a junior synonym for *M. variegata*. Nartshuk (2006) described the new species *M. meigeni* based on the absence of the holotype *M. laeta* though pointing out, that *M. meigeni* may be identical to the previously described *M. laeta* (Nartshuk 2006, Nartshuk and Fedoseeva 2011). In the key to species of the genus (Nartshuk and Fedoseeva 2010), *M. laeta* is replaced by *M. meigeni*. However, we think that the species-specific description presented by the aforementioned authors (shape of the aedeagus and anterior process of the postgonite) is not sufficiently reliable to consider *M. meigeni* as a new species. Despite the description of *M. meigeni* by Nartshuk as a species unique to Slovenia, the shape of the aedeagus and anterior process of the postgonite, the key features of this species, are similar to those in *M. laeta* which was described as a species by different authors (Meigen 1830, 1838, Fedoseeva 1960, Hubicka 1970) from numerous regions of Europe. In our opinion, it suggests possible regional variability of *M. laeta* for a number of key features. We propose to go back to the traditional name of the species previously defined as *M. laeta* (Meigen 1830, 1838).

Molecular-genetic analysis of the CO1 mtDNA gene revealed a concordance between the haplotype and size of the postgonite anterior process (Safonkin et al. 2016); the species closer to the hypothetical ancestors of the cluster demonstrate the largest size of the anterior process of the postgonite. As stated in the above paper, the cluster in question was named as “meigeni”, since the Network (phylogenetic program) places *M. meigeni* closer to a hypothetical haplotype than *M. variegata*. The dendrogram displays *M. meigeni* and *M. variegata* as practically equidistant from the hypothetical haplotype (Fig. 1). Also, the area of the anterior process of the postgonite in *M. variegata* is significantly larger than in *M. meigeni* (Table 2). Based on the concordance between molecular phylogenetic results (Fig. 1) and the size of the postgonite anterior process, we propose to name the cluster in question as “variegata” instead of “meigeni”. The cluster’s name is taken from the name of the species closer to the ancestor haplotype and with the largest size of the anterior process of the postgonite.

Thus, it is possible to identify seven species in the “variegata” cluster according to a combination of external key features and the postgonite structure: *M. rufa*, *M. bohemica*, *M. femorata*, *M. laeta*, *M. variegata*, *M. mosquensis*, and *M. elbergi*. Based on the original description (Nartshuk 1992), *M. zimzerla* Nartshuk, 1992 can be also placed into the “variegata” cluster. Molecular analysis of the second part of the CO1 mtDNA gene confirms the identification of six selected species of the “variegata” cluster. Currently, there is no molecular analysis data for *M. elbergi* and *M. zimzerla*, though the key features of *M. elbergi* are close to those in species of the “variegata” cluster, but the original description of *M. zimzerla* puts this species close to *M. variegata*, one of the species with large inter-population variability. Nartshuk and Fedoseeva (2011) pointed out that *M. variegata* sensu Fedoseeva, 1960 = *M. zimzerla* Nartshuk, 1992.

Our comparative analysis showed a high degree of variability of external key features among species of the “variegata” cluster. The structure of the postgonite and, especially, the size of its anterior process is species specific in males, whereas the females cannot be reliably identified as particular species only by external morphology. For example, in the keys of Fedoseeva (2003) and Nartshuk and Fedoseeva (2010), the presence of a ‘large number’ of black setae on the lower surface of the genae is the main criterion in identification of *M. bohemica*. Based on our analysis of the populations of this species, only one-fifth to one-third of female specimens can be identified by this character as *M. bohemica*.

The same is true of *M. meigeni*; the main diagnostic feature in the identification of this species is the mid stripe of the mesonotum which reaches the scutellum but does not pass through it. However, in 1.4–29% of individuals of *M. bohemica* populations the mid stripe reaches the shield, instead. Also, the color of the stripes of the mesonotum varies from light to dark among specimens of *M. bohemica* populations. Such an important feature for species identification of the “variegata” cluster as palp color, can vary considerably from light to dark. In addition, the species are divided into two groups based on the shape of the palpi. However, the analysis of the shape of the palp requires examination of the object from the same angle, which is not always feasible when using the dry specimens.

**Figure 2.**
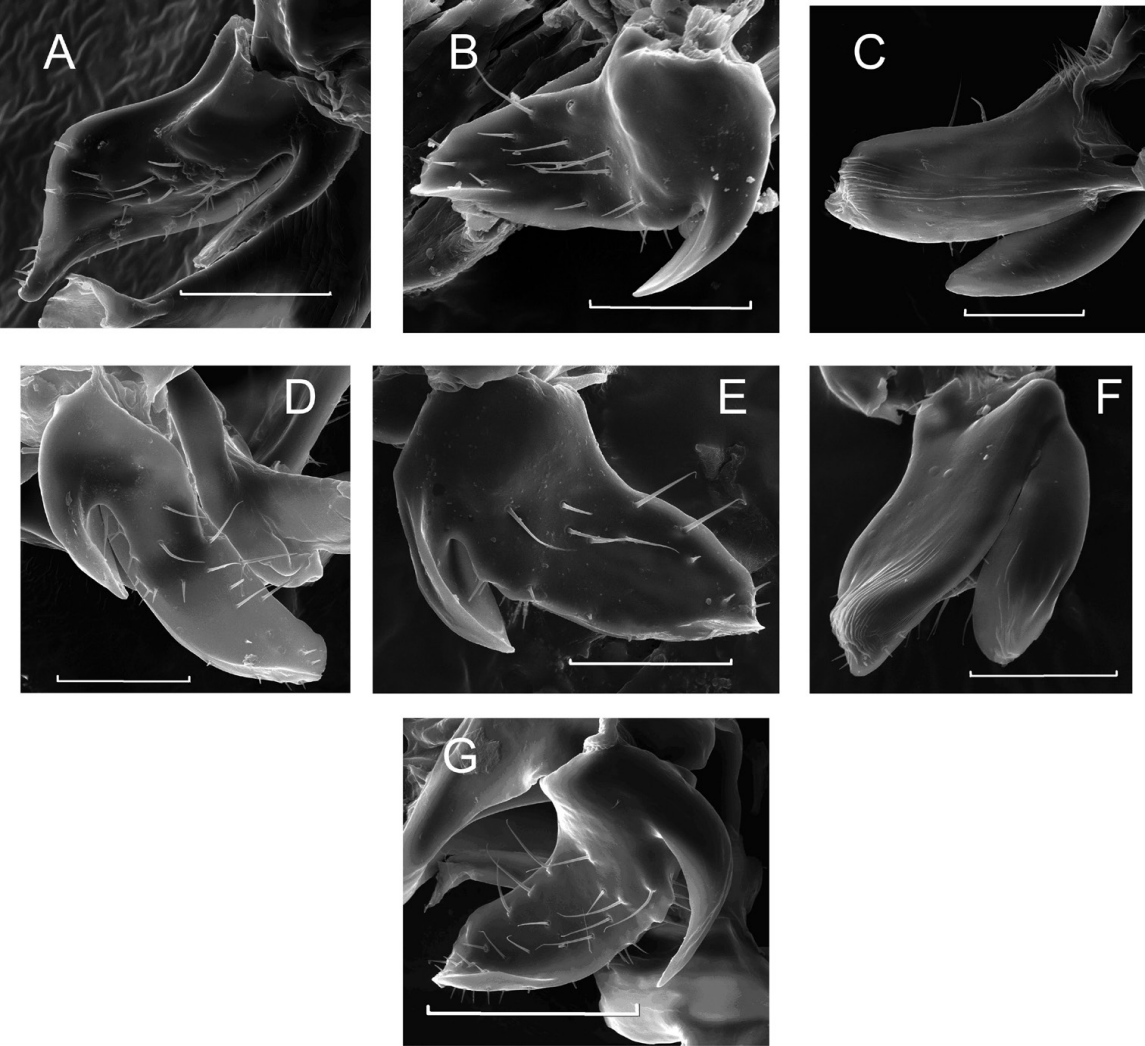
The postgonites of *M. bohemica* (**A**), *M. elbergi* (**B**), *M. femorata* (**C**), *M. laeta* (**D**), *M. mosquensis* (**E**), *M. rufa* (**F**), *M. variegata* (**G**). Scale bar: 50 μm.

## Conclusions

We suggest, that approaches to the identification of the “variegata” cluster species should include external morphological and dimensional features of both males and females. Additionally, the analysis of males by peculiarities of the postgonites (structure and size of the area of the anterior process of the postgonite) and the analysis of females by the CO1 mtDNA should be used. This will allow the reliable species identification. A similar approach should be used in the identification of adults in other clusters of the genus *Meromyza*.

### Key to species included in the proposed cluster “variegata”

**Table:** 

1	Palpi strongly darkened	**2**
–	Palpi light, slightly darkened on the top (up to 1/2 height)	**4**
2	Black setae on the lower surface of genae (postgonite; Fig. 2B)	***M. elbergi***
–	Without black setae on the lower surface of genae	**3**
3	Hind femurs heavily thickened, postgonite area, body size, height of frontal triangle, height of genae relative to the 3rd segment of the pedicel is large, the end of the projecting part of anterior process of postgonite with transverse folds, posterior process of the postgonite is adjacent to the anterior one from the back (Fig. 2C)	***M. femorata***
–	Hind femur thinner, postgonite area, body size, height of frontal triangle, height of genae relative to the 3rd segment of the pedicel is less, the end of projecting part of anterior process of postgonite is round, posterior and anterior processes of postgonite are fused (Fig. 2D) ***M. laeta***
4	Sampling of specimens with black setae on the lower surface of genae. The length of mesonotum is 3-fold over the scutellum length (postgonite; Fig. 2A)	***M. bohemica***
–	Without black setae on the lower surface of genae. Mesonotum is 3.1–3.4-fold over the scutellum.	**5**
5	Dark occiput spot, mid strip of mesonotum passes through scutellum, small height of frontal triangle (0.31 mm) (postgonite; Fig. 2E)	***M. mosquensis***
–	without dark occiput spot and the strip on the scutellum, large height of frontal triangle (0.32–0.38 mm)	**6**
6	Color of the strips of mesonotum rusty-red, yellow-brown, sometimes brownish, small genae height relative to the 3rd segment of the pedicel and small length of the mesonotum. Wide posterior process of the postgonite (Fig. 2F)	***M. rufa***
–	Color of the stripes from brown to black, large height of genae relative to the 3rd segment of the pedicel and the length of the mesonotum. Acuminate curved posterior process of the postgonite (Fig. 2G)	***M. variegata***
